# The Non-Tightness of a Convex Relaxation to Rotation Recovery

**DOI:** 10.3390/s21217358

**Published:** 2021-11-05

**Authors:** Yuval Alfassi, Daniel Keren, Bruce Reznick

**Affiliations:** 1 Department of Computer Science, University of Haifa, Haifa 3498838, Israel; yuvalalfassi@gmail.com; 2 Department of Mathematics, University of Illinois at Urbana-Champaign, Urbana, IL 61801, USA; reznick@illinois.edu

**Keywords:** PNP, rotation recovery, convex relaxation, polynomial optimization

## Abstract

We study the Perspective-n-Point (PNP) problem, which is fundamental in 3D vision, for the recovery of camera translation and rotation. A common solution applies polynomial sum-of-squares (SOS) relaxation techniques via semidefinite programming. Our main result is that the polynomials which should be optimized can be non-negative but not SOS, hence the resulting convex relaxation is not tight; specifically, we present an example of real-life configurations for which the convex relaxation in the Lasserre Hierarchy fails, in both the second and third levels. In addition to the theoretical contribution, the conclusion for practitioners is that this commonly-used approach can fail; our experiments suggest that using higher levels of the Lasserre Hierarchy reduces the probability of failure. The methods we use are mostly drawn from the area of polynomial optimization and convex relaxation; we also use some results from real algebraic geometry, as well as Matlab optimization packages for PNP.

## 1. Introduction

The Perspective-n-Point (PNP) problem is a cornerstone of 3D computer vision [1,2,3,4,5]. We deal with its most fundamental variant: given 2D projections of 3D points whose real-world coordinates $p_{i}={\left(x_{i},y_{i},z_{i}\right)}_{i=1}^{n}$ are known, one seeks to determine the camera position and angle, i.e., a translation vector *T* and rotation matrix *R*, relative to the world coordinate frame, which provide an optimal fit between the 3D points and their projections on the camera image plane. We prove here a *negative* result in terms of complexity: specifically, that a commonly-used approach for convex relaxation of the problem fails for an open subset in the configuration space. A concrete example of such a configuration is provided. On the positive side, we note that, as the levels of the convex relaxation increase, the probability of failure decreases.

### The PNP Problem: Details

Denoting by {*u_i_*} the unit vectors in the direction of the projections of {*p_i_*}, we obtain the very well-known expression to minimize, as a function of *T* and *R*:

(1) $$\sum_{i}{\left\|Q_{i}(Rp_{i}+T)\right\|}^{2},Q_{i}≜I-u_{i}u_{i}^{t}$$

**Figure 1:**
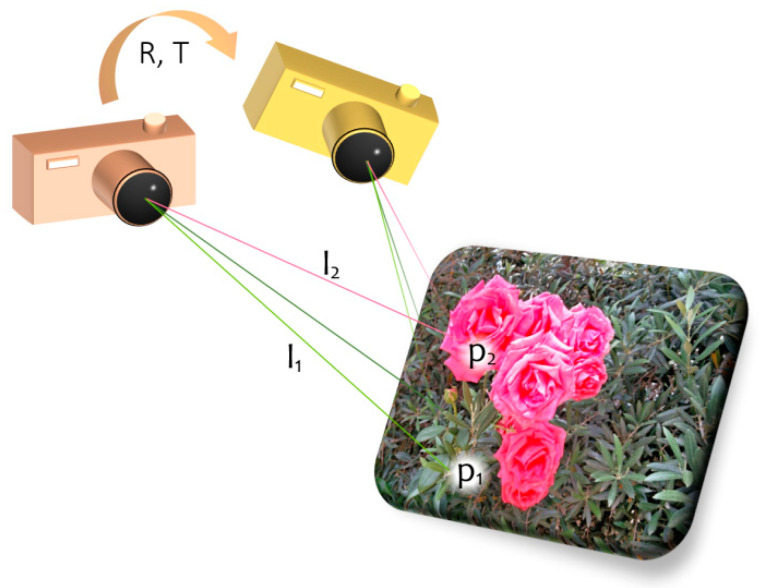
Illustration of the PNP problem.

Geometrically (see Figure 1), this means rotating and translating the point set {*p_i_*}, so that the sum of squared norms of their respective projections on the lines spanned by the {*u_i_*} is maximized (i.e., the points are optimally aligned with the respective “lines of sight”). Due to the great importance of this problem in real-life applications, it was addressed in numerous papers. The solution obtained by minimizing Equation (1) is global and does not rely on approximations and iterative schemes.

The minimization proceeds by first solving for *T* by differentiation, and then substituting back into Equation (1). If the rotation matrix *R* is “flattened” and viewed as a vector of length 9, we obtain the following minimization problem over rotation matrices (details are described in [2], see also a general survey on optimization of quadratic functions over rotation groups in [6]):

(2) $$\mathrm{Minimize}\;R^{t}PR,\;P=\underset{i}{\sum }\left(C_{i}^{t}-A^{t}\right)Q_{i}\left(C_{i}-A\right),A≜{\left(\underset{i}{\sum }Q_{i}\right)}^{-1}\left(\underset{i}{\sum }Q_{i}C_{i}\right)$$

where:

$$C_{i}≜\left(\begin{array}{ccccccccc} x_{i} & y_{i} & z_{i} & 0 & 0 & 0 & 0 & 0 & 0\\ 0 & 0 & 0 & x_{i} & y_{i} & z_{i} & 0 & 0 & 0\\ 0 & 0 & 0 & 0 & 0 & 0 & x_{i} & y_{i} & z_{i} \end{array}\right)$$

Note that since $Q_{i}^{2}=Q_{i}$ and $Q_{i}^{t}=Q_{i},$ *P* is positive semidefinite. To minimize this quadratic polynomial over the space of rotation matrices, it is customary to represent *R*’s elements as quadratics in the four components of a unit quaternion [7]:

(3) $$\begin{array}{l} R=[q_{1}^{2}+q_{2}^{2}-q_{3}^{2}-q_{4}^{2},2q_{2}q_{3}+2q_{1}q_{4},2q_{2}q_{4}-2q_{1}q_{3},\\ 2q_{2}q_{3}-2q_{1}q_{4},q_{1}^{2}+q_{3}^{2}-q_{2}^{2}-q_{4}^{2},2q_{3}q_{4}+2q_{1}q_{2},\\ 2q_{2}q_{4}+2q_{1}q_{3},2q_{3}q_{4}-2q_{1}q_{2},q_{1}^{2}+q_{4}^{2}-q_{2}^{2}-q_{3}^{2}] \end{array}$$

Plugging this representation into Equation (2) yields a positive semidefinite quartic homogenous polynomial in *q*_1_, *q*_2_, *q*_3_, *q*_4_, which should be minimized under the additional constraint $q_{1}^{2}+q_{2}^{2}+q_{3}^{2}+q_{4}^{2}=1$. Since this polynomial depends on the input, {*p_i_*} and {*u_i_*}, it will be denoted by $P_{\left\{p_{i},u_{i}\right\}}\left(q\right)$, where $q≜\left(q_{1},q_{2},q_{3},q_{4}\right)$. We shall say that a polynomial is *realizable in PNP* iff it is equal to $P_{\left\{p_{i},u_{i}\right\}}\left(q\right)$ for some {*p_i_*} and {*u_i_*}. We add the common requirements that, for every *i,* the *z*-coordinate of {*p_i_*} is non-negative, and 〈*p_i_*,*u_i_*〉 ≥ 0; these requirements correspond to the physical scenario of a forward-looking camera, i.e the field of view is limited to one side of the image plane.

The dependence of the 35 coefficients of $P_{\left\{p_{i},u_{i}\right\}}\left(q\right)$ on {*p_i_*} and {*u_i_*} is very complicated, due to the operations of matrix inversion and multiplication in Equation (2). While for given values of {*p_i_*} and {*u_i_*} it is straightforward to compute $P_{\left\{p_{i},u_{i}\right\}}\left(q\right)$, the question of characterizing the realizable polynomials is quite subtle, and we are not aware of a general solution to it. 

Summarizing, the PNP problem becomes one of minimizing a homogenous quartic polynomial over $S^{3}=\left\{q\in {ℜ}^{4}\left|\;\right|\left|q\right||=1\right\}$. However, there is no closed-form solution to this problem, as it requires finding the minimal value of a fourth-degree homogeneous quartic in four variables, which are constrained to lie on *S*^3^ (i.e., the sum of their squares must be equal to 1). While the global minimum is known to exist, since the target function is continuous and *S*^3^ is a compact set, recovering the minimum is a notoriously difficult problem [8,9]. In principle it can be solved using Lagrange multipliers, which reduce the optimization to the solution of a polynomial system, but this solution is lengthy, applies numerical techniques, and often multiple solutions are present, which need to be pruned (in addition to removing complex solutions); please see Appendix A for the details.

These difficulties have led practitioners [2,4] to apply a very well-developed and popular method for polynomial optimization, known as *sum of squares* (*SOS*) *relaxation* [8,9,10], which we now briefly describe.

## 2. Previous Work and Methods

Clearly, the problem

$$\underset{q\in S^{3}}{\arg\min}\;p(q)$$

is equivalent to the problem

$$\underset{\gamma \in R}{\arg\max}\;\mathrm{such}\mathrm{that}p(q)-\gamma \geq 0\;\mathrm{for}\mathrm{all}\;q\in S^{3}$$

That is, the minimal value *p*(*q*) attains on *S*^3^ is equal to the maximal value of *γ* such that the polynomial *p*(*q*) − *γ* is non-negative on $S^{3}$. Thus, the task of finding *γ* can be expressed as the problem of determining whether a polynomial is non-negative on $S^{3}$. If there was a mechanism that allows to quickly determine whether a polynomial has this property, it could have been used (possibly combined with a binary search on *γ*) to find the minimum.

Alas, determining whether a polynomial is non-negative (even on a compact set) is notoriously difficult [8,9,10]. Therefore, a great deal of work concentrated on a sub-family of positive polynomials—which are defined by the property that they can be written as a *sum of squares* (SOS) of other polynomials (hence are clearly non-negative). The class of SOS polynomials has an important advantage: the question of whether a homogeneous polynomial of degree d in n variables is an SOS can be cast and solved as a semidefinite programing (SDP) problem, on matrices of size $O\left(n^{d}\right)$ [8,10].

The question whether every non-negative polynomial is SOS was first studied by Hilbert [11], who was able to prove the existence (but not an explicit construction) of non-negative, non-SOS polynomials. Much later, some simple and beautiful examples of such polynomials were found, one of them which we will use in this paper.

**Lemma** **1** ([12])**.** *Denote* $b\left(q_{1},q_{2},q_{3},q_{4}\right)=q_{1}^{2}q_{2}^{2}+q_{1}^{2}q_{3}^{2}+q_{2}^{2}q_{3}^{2}+q_{4}^{4}-4q_{1}q_{2}q_{3}q_{4}$*. Then b is non-negative but is not an SOS.*

The set of non-negative, non-SOS quartic polynomials in four variables is denoted $\Delta_{4,4}$, and it is known to be open (in the natural topology in coefficient space). This means that if a polynomial is in $\Delta_{4,4}$, then small enough perturbations of its coefficient will define polynomials that are also in $\Delta_{4,4}$.

To apply SOS polynomials to the minimization of polynomials over a sphere (similar results hold for more general algebraic varieties, but are more complicated and will not be described here), the following result is often applied:

**Theorem** **1** ([13])**.** *Assume that a polynomial* p(q) *is positive on* $S^{3}$*. Then there exist polynomials* ϕ(q) *and* s(q) *such that* $\;p\left(q\right)=\left({\left\|q\right\|}^{2}-1\right)\phi \left(q\right)+s\left(q\right)$*, where* s(q) *is an SOS polynomial.*

Note that if such polynomials exist, then, since on $S^{3}$, ${\left\|q\right\|}^{2}-1=0$, it holds that on $S^{3}$, p(q)=s(q), hence p(q) is non-negative. This is often referred to as a *certificate of positivity* for p(q) on $S^{3}$. However, the theorem does not guarantee a bound on the degrees of the “auxiliary” polynomials ϕ(q) and s(q). For example, if the degree of p(q) is 4, then it may well be that the degree of ϕ(q) is 4 and the degree of s(q) is 6 (i.e., it is the sum of squares of cubic polynomials). In that case, the terms of degrees 5 and 6 will cancel out in $\left({\left\|q\right\|}^{2}-1\right)\phi \left(q\right)$ and s(q).

Since the degrees of ϕ(q) and s(q) are not known in advance, optimization is carried out in the framework of the *Lasserre hierarchy* [10] for successively approximating the minimum. In our case, the levels of the hierarchy are defined as follows: setting deg(s)=2t (clearly, the degree of an SOS polynomial is even), defines the *t*-th level. As the levels increase, the minimum approaches the correct one, alas the computational complexity rapidly increases with t, since the size of the matrices to optimize over in the corresponding semidefinite programming problem is of order $4^{t}\times 4^{t}$. The quest for a fast solution led researchers to implement the case t=2 (i.e., the second level), which runs in reasonable time, and noting that, *empirically, it appears to work well* [2,4]. Still, the question remains: can this approach run into cases in which the second level fails to find the minimum (i.e., it is not tight)? We answer this question in the affirmative. To the best of our knowledge, we provide the first concrete example of a PNP configuration leading to a polynomial which fails the second Lasserre level. We then provide an example which also fails the third level. 

### Other Work on Convex Relaxation for Rotation Recovery

Recently, the question whether convex relaxation for rotation recovery (and other problems in computer vision) is tight, was addressed. In [14], the following question is studied: given an initial value at which the relaxation is tight, is there a neighborhood of this initial value at which tightness holds? (here, we empirically study the *opposite* scenario—i.e., how stable under additive noise is the “bad” property of the relaxation *not* being tight. In [15], an improved Lagrangian dual relaxation is provided, and it is noted that *empirically*, its performance is excellent, i.e the relaxation is tight. The work which we found to be closest to ours in spirit is [6], in which the question as to when the convex relaxation is tight is directly addressed, both for recovery of a single and multiple rotations. The authors note that, empirically (while not theoretically!), the convex relaxation for the recovery of a single rotation is tight, as opposed to the case of two rotations. Then, a general study of the tightness of convex relaxations is undertaken, relying in part on deep results from algebraic geometry, notably a study of minimal varieties [16].

Interestingly, the results in [6,15] suggest that the example we provide here, for the failure of convex relaxation for single rotation recovery, is rare; alas, it is not singular (i.e, the set of points-lines configurations in which this failure occurs are of full dimension, which immediately follows since $\Delta_{4,4}$ is an open set). An interesting question, which we hope to pursue in the future, is to estimate “how rare” it is, in terms of probability of failure. This question is related to estimating how many positive polynomials are not SOS [17]; while asymptotic results exist, we are not aware of research on this question for small numbers of variables and low degrees.

## 3. Results

We now present the results, which consist both of theoretical analysis and experiments which support them.

### 3.1. Main Result: The Inadequacy of the Second Lasserre Level for Solving the PNP Problem

Recall the polynomial $b\left(q_{1},q_{2},q_{3},q_{4}\right)=q_{1}^{2}q_{2}^{2}+q_{1}^{2}q_{3}^{2}+q_{2}^{2}q_{3}^{2}+q_{4}^{4}-4q_{1}q_{2}q_{3}q_{4}$ which is non-negative and also non-SOS. For any scalar c>0, denote

$$b_{c}\left(q_{1},q_{2},q_{3},q_{4}\right)=b\left(q_{1},q_{2},q_{3},q_{4}\right)+c{\left\|q\right\|}^{4}$$

**Lemma** **2.** *The minimum of* $b_{c}\left(q_{1},q_{2},q_{3},q_{4}\right)$ *on* $S^{3}$ *cannot be recovered in the second Lasserre level.*

**Proof.**  Since there are points on *S*^3^ for which $b\left(q_{1},q_{2},q_{3},q_{4}\right)=0$, the minimum of $b_{c}\left(q_{1},q_{2},q_{3},q_{4}\right)$ on $S^{3}$ is c. But the following identity, which is required for obtaining the solution in the second Lasserre level, cannot hold for a quartic SOS s(q):

(4) $$b\left(q\right)+c{\left\|q\right\|}^{4}-c=\left({\left\|q\right\|}^{2}-1\right)\phi \left(q\right)+s\left(q\right)$$

To see this, note that when Equation (4) is restricted to $S^{3}$, it becomes $b\left(q\right)=s\left(q\right)=\underset{i}{\sum }p_{i}^{2}\left(q\right)$, where $p_{i}\left(q\right)$ are quadratics in q (since s(q) is a quartic SOS). Denote $p_{i}\left(q\right)=p_{2000}q_{1}^{2}+\ldots +p_{0000}$ (i is suppressed since the following holds for every $p_{i}\left(q\right)$). Obviously, if $q_{0}\in S^{3}$ and $b\left(q_{0}\right)=0$, then $p_{i}\left(q_{0}\right)=0$. There are 14 points on $S^{3}$ at which $b\left(q_{0}\right)=0$: (±1,0,0,0),(0,±1,0,0),(0,0,±1,0), as well as all combinations of the form $\left(\frac{1}{2}\right)\left(\pm 1,\pm 1,\pm 1,\pm 1\right)$ in which an even number of the coordinates are positive. Substituting these 14 points in $p_{2000}q_{1}^{2}+\ldots +p_{0000}$, equating to 0 and solving, yields that every $p_{i}\left(q\right)$ must be of the form $a_{i}\left({\left\|q\right\|}^{2}-1\right)+r_{i}\left(q\right)$, where $r_{i}\left(q\right)$ is a *quadratic form* (i.e has no linear and constant terms). Therefore, it must hold that $b\left(q\right)=\underset{i}{\sum }a_{i}^{2}{\left({\left\|q\right\|}^{2}-1\right)}^{2}+2\left({\left\|q\right\|}^{2}-1\right)r_{i}\left(q\right)+r_{i}^{2}\left(q\right)$. Restricting to $S^{3}$ again, $b\left(q\right)=\underset{i}{\sum }r_{i}^{2}\left(q\right)$, but now both sides of the identity are homogenous quartics. Since they are equal on $S^{3}$, they must be equal everywhere, but *b*(*q*) is not an SOS, a contradiction. □

**Note:** The proof depends on the fact that b(q) is not an SOS polynomial. To see this, observe that if s is a quartic SOS, then the following identity holds: $s+c{\left\|q\right\|}^{4}-c=\left({\left\|q\right\|}^{2}-1\right)\left(c{\left\|q\right\|}^{2}+c\right)+s$.

In order to prove that an instance of the PNP problem can also fail in the second Lasserre level, we need:

**Lemma** **3.** *There exists a PNP configuration of 3D points,*$\left\{p_{i}\right\}$*, and their lines of projection on the camera image plane, *$\left\{u_{i}\right\}$*, such that*${Ρ}_{\left\{p_{i},u_{i}\right\}}\left(q\right)=b_{c}\left(q_{1},q_{2},q_{3},q_{4}\right)$*for some*c>0.

**Proof.**  The proof is constructive, and consists of an example of $\left\{p_{i}\right\}$, $\left\{u_{i}\right\}$, which yield $b_{c}\left(q_{1},q_{2},q_{3},q_{4}\right)$. To find such $\left\{p_{i}\right\}$, $\left\{u_{i}\right\}$, the following paradigm was adopted:

(1) Fix a certain value of c. This yields a polynomial $b_{c}\left(q_{1},q_{2},q_{3},q_{4}\right)$ with fixed scalar coefficients.
(2) Define a distance between two polynomials f,g as the sum of squares of differences of their coefficients. In our case, f,g are homogenous quartics, hence there are 35 terms in this sum (the number of coefficients).
(3) For any choice of $\left\{p_{i}\right\}$, $\left\{u_{i}\right\}$, define the function $E\left(\left\{p_{i},u_{i}\right\}\right)$ as the distance between ${Ρ}_{\left\{p_{i},u_{i}\right\}}\left(q\right)$ and $b_{c}\left(q_{1},q_{2},q_{3},q_{4}\right)$.
(4) Find $\left\{p_{i}\right\}$, $\left\{u_{i}\right\}$ which minimize $E\left(\left\{p_{i},u_{i}\right\}\right)$.

We note that the optimization problem 4 above is rather difficult, as the relation between $\left\{p_{i}\right\}$, $\left\{u_{i}\right\}$ and the coefficients of ${Ρ}_{\left\{p_{i},u_{i}\right\}}\left(q\right)$ is very complicated. Even when setting the vectors $\left\{u_{i}\right\}$ to scalar values, each of the 35 coefficients is a quadratic polynomial in the 3n variables ${\left\{\left(x_{i},y_{i},z_{i}\right)\right\}}_{i=1}^{n}$, i.e., its size is $\left(\begin{array}{c} 3n\\ 2 \end{array}\right)$. After summing the squares of all these expressions with the respective coefficients of $b_{c}\left(q_{1},q_{2},q_{3},q_{4}\right)$ subtracted, the resulting expression to be minimized is a quartic in the 3n variables ${\left\{\left(x_{i},y_{i},z_{i}\right)\right\}}_{i=1}^{n}$, hence its size is $O\left(n^{4}\right)$. The most general expression—with non-scalar values for the vectors $\left\{u_{i}\right\}$—has the same structure, but with each of the $O\left(n^{4}\right)$ coefficients being a very complicated rational function of the components of all the $u_{i}’s$, resulting from the matrix inversion and multiplication operations in Equation (2).

Therefore, in order to obtain a feasible minimization problem, we have applied the following methodology:

- Chosen n=14.
- Set the $\left\{u_{i}\right\}$ to fixed scalar values, by evenly distributing them over the upper half of the unit sphere.
- Minimized $E\left(\left\{p_{i},u_{i}\right\}\right)$ only over $\left\{p_{i}\right\}$. Note that even then, no closed-form solution exists; however, the *Optimization* routine of the Maple© software package (Waterloo Maple Inc., 615 Kumpf Dr, Waterloo, ON N2V 1K8, Canada) was able to solve this problem with very high accuracy, obtaining a minimal value of $E\left(\left\{p_{i},u_{i}\right\}\right)$ roughly equal to $10^{-18}$.
- Empirically, it turned out that a solution exists only for values of c larger than a certain threshold. Since our goal in this short submission is only to demonstrate that real PNP scenarios can fail the second Lasserre level, we did not attempt to find the smallest values of c and n; however, these are interesting problems which merit further study.
  In order to verify the correctness of the optimization, the resulting values of $\left\{p_{i}\right\}$, $\left\{u_{i}\right\}$ have been plugged into two packages for solving the PNP problem using SOS relaxation, and have indeed caused them to fail (see “Experimental Results”).

□

### 3.2. Some Comments on the Result

- The long running time required to produce the configuration which yields $b_{c}\left(q_{1},q_{2},q_{3},q_{4}\right)$ is not an issue, as our goal was to show that such a configuration exists.
- The configuration is *stable,* i.e small perturbations in the $\left\{p_{i}\right\}$, $\left\{u_{i}\right\}$ still yield a polynomial which fails the relaxation. This is due to the fact that the set $\Delta_{4,4}$ is *open* [18]. That is, the “problematic” configurations are not restricted to a low-dimensional manifold.
- Note that a rotation of $\left\{p_{i}\right\}$ and $\left\{u_{i}\right\}$ would have yielded a different polynomial, which would have also failed the relaxation; further, it would have been different from $b_{c}\left(q_{1},q_{2},q_{3},q_{4}\right)$. Thus, the problem the configuration poses cannot be solved by checking whether the resulting polynomial is equal to $b_{c}\left(q_{1},q_{2},q_{3},q_{4}\right)$.
- While, as proved in the following section, the *third* Lasserre level can solve the PNP configuration which yield $b_{c}\left(q_{1},q_{2},q_{3},q_{4}\right)$, the solution did not converge due to numerical instabilities; a solution was obtained only at the fourth level. The numerical instability of solving optimization problems involving polynomials which are positive but not SOS was noticed by other researchers [9]. From the practical point of view, this indicates that there are PNP configurations at which higher Lasserre levels are required for the solution, than those indicated by Lemmas 3 and 5.
- One may wonder why the expression $c{\left\|q\right\|}^{4}$ is added to $b\left(q_{1},q_{2},q_{3},q_{4}\right)$. The reason is that we could not realize $b\left(q_{1},q_{2},q_{3},q_{4}\right)$ in PNP, since $b\left(q_{1},q_{2},q_{3},q_{4}\right)$ assumes the value of 0 at 14 points on $S^{3}$. This means that to realize it in PNP, the matrix P in Equation (2) would have had to be highly singular.

### 3.3. PNP and the Third Lasserre Level

It turns out that a PNP configuration which yields $b_{c}\left(q_{1},q_{2},q_{3},q_{4}\right)$ can *theoretically* be solved in the next Lasserre level—the third one. To prove this, we must produce a quartic polynomial ϕ(q) and a sextic SOS polynomial s(q) satisfying $b\left(q\right)+c{\left\|q\right\|}^{4}-c=\left({\left\|q\right\|}^{2}-1\right)\phi \left(q\right)+s\left(q\right)$. Noting that:$b\left(q\right)+c{\left\|q\right\|}^{4}-c=\left({\left\|q\right\|}^{2}-1\right)\left(c{\left\|q\right\|}^{2}+c-b\left(q\right)\right)+{\left\|q\right\|}^{2}b\left(q\right)$, it remains to prove that ${\left\|q\right\|}^{2}b\left(q\right)$ is an SOS. This can be proved by directly verifying that:

$$\begin{array}{l} 6{\left\|q\right\|}^{2}b(q)=6{\left(w^{3}-xyz\right)}^{2}+{\left(-w^{2}x-3wyz+2xy^{2}+2xz^{2}\right)}^{2}+\\ {\left(-w^{2}y-3wxz+2x^{2}y+2yz^{2}\right)}^{2}+{\left(-w^{2}z-3wxy+2x^{2}z+2y^{2}z\right)}^{2}\\ +2x^{2}{\left(y^{2}-z^{2}\right)}^{2}+2y^{2}{\left(x^{2}-z^{2}\right)}^{2}+2z^{2}{\left(x^{2}-y^{2}\right)}^{2}+5w^{2}{\left(wx-yz\right)}^{2}+\\ 5w^{2}{\left(wy-xz\right)}^{2}+5w^{2}{\left(wz-xy\right)}^{2} \end{array}$$

(where in order to reduce equation clutter, we denote q=(x,y,z,w)).

It turns out that for our problem, the properties of being solvable in the third Lasserre level and of $p\left(q\right){\left\|q\right\|}^{2}$ being an SOS are equivalent. This is summarized in the following:

**Lemma** **4.** *Assume that *p(q)*is a homogenous quartic which is non-negative on S^3^ and, in addition, its minimum on *$S^{3}$*is equal to 0. Assume further that the minimum of*p(q)*on*$S^{3}$*can be obtained in the third Lasserre level. Then the polynomial *$p\left(q\right){\left\|q\right\|}^{2}$*must be an SOS*.

**Proof.**  Assume that the minimum can be obtained in the third Lasserre level. Then, there is a quatric ϕ(q) and a sextic SOS s(q), such that the following holds:

(5) $$p\left(q\right)+c{\left\|q\right\|}^{4}-c=\left({\left\|q\right\|}^{2}-1\right)\phi \left(q\right)+s\left(q\right)$$

Next, we write down Equation (5), but while separating ϕ(q) and s(q) to homogeneous forms (in order to reduce equation clutter, q is suppressed wherever possible, as all polynomials are in *q*):

(6) $$p+c{\left\|q\right\|}^{4}-c=\left({\left\|q\right\|}^{2}-1\right)\left(\phi_{4}+\phi_{3}+\phi_{2}+\phi_{1}+\phi_{0}\right)+\overset{k}{\underset{i=1}{\sum }}{\left(C_{i}+Q_{i}+L_{i}+A_{i}\right)}^{2}$$

That is, $\phi_{l}$ contains only terms of total degree l, and $C_{i},Q_{i},L_{i},A_{i}$ stand for cubic, quadratic, linear, and constant terms in the polynomials whose squares compromise s(q).

Since two polynomials are equal iff all their respective forms are equal, Equation (6) yields the following identities for the even-degree forms:

Degree 0: $-c=-\phi_{0}+\underset{i}{\sum }A_{i}^{2}$

Degree 2: $0={\left\|q\right\|}^{2}\phi_{0}-\phi_{2}+\underset{i}{\sum }L_{i}^{2}+2\underset{i}{\sum }A_{i}Q_{i}$

Degree 4: $p+c{\left\|q\right\|}^{4}={\left\|q\right\|}^{2}\phi_{2}-\phi_{4}+\underset{i}{\sum }Q_{i}^{2}+2\underset{i}{\sum }L_{i}C_{i}$

Degree 6: $0={\left\|q\right\|}^{2}\phi_{4}+\underset{i}{\sum }C_{i}^{2}$

Extracting $\phi_{4},\phi_{2},\phi_{0}$, substituting in the equation for degree 4 above, and multiplying by ${\left\|q\right\|}^{2}$, yields: ${\left\|q\right\|}^{2}p\left(q\right)=\underset{i}{\sum }{\left(C_{i}+L_{i}{\left\|q\right\|}^{2}\right)}^{2}+{\left\|q\right\|}^{2}\underset{i}{\sum }{\left(Q_{i}+A_{i}{\left\|q\right\|}^{2}\right)}^{2}$, which is a sum of squares. □

As a consequence of the above, the question of whether the PNP problem can also fail to have a solution in the third Lasserre level is equivalent to the question whether there exists a polynomial  p(q) such that the following hold:

- p(q) is non-negative on $S^{3}$, and its minimum on $S^{3}$ is 0.
- $p\left(q\right)+c{\left\|q\right\|}^{4}$ is realizable in PNP for some c>0.
- $p\left(q\right){\left\|q\right\|}^{2}$ is *not* an SOS polynomial.

**Lemma** **5.** *The answer to the above is affirmative.*

**Proof.**  Define $p\left(q\right)=b\left(q_{1},q_{2},q_{3},\frac{q_{4}}{5}\right)$. As for $b\left(q\right)+c{\left\|q\right\|}^{4}$, direct optimization proves that $p\left(q\right)+c{\left\|q\right\|}^{4}$ is realizable in PNP for c=2. Also, $p\left(q\right){\left\|q\right\|}^{2}$ is not an SOS, as can be verified directly by applying dedicated software. □

More generally:

**Lemma** **6.** $b\left(q_{1},q_{2},q_{3},\frac{q_{4}}{r}\right)$*is not an SOS for* $r>\sqrt{6}$ [19,20,21].

As noted in Section 2, the complexity of solving the SOS problem quickly increases with the Lasserre level, as its solution requires processing matrices of size $O\left(4^{t}\right)$. On the average, running time (on an Intel(R) Core^TM^ i5-1135G7 CPU @ 2.40 GHz) were 0.07, 0.196, 0.98, 5.275 and 27.02 s, respectively for levels 2, 3, 4, 5 and 6. 

### 3.4. Experimental Results

**Figure 2:**
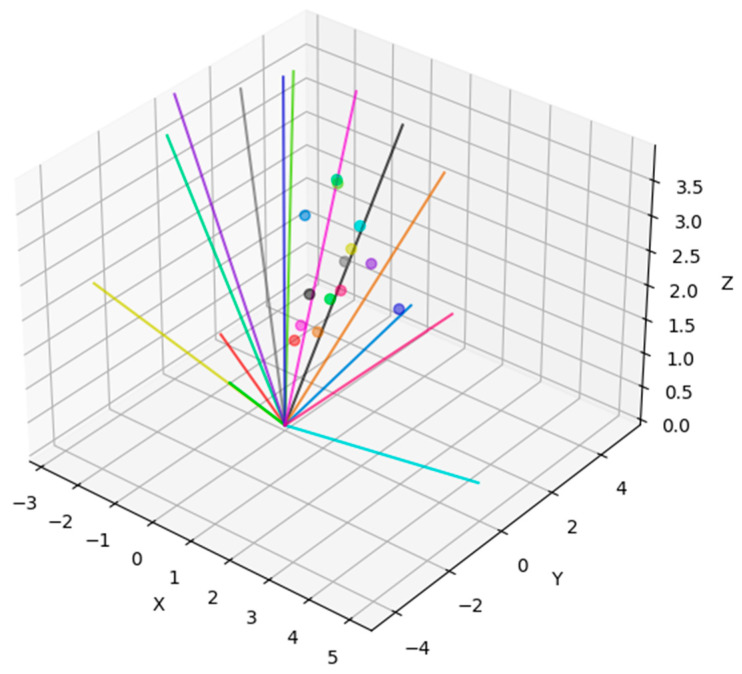
PNP scenario which fails the second Lasserre level (same colors for line-point pairs).

**Figure 3:**
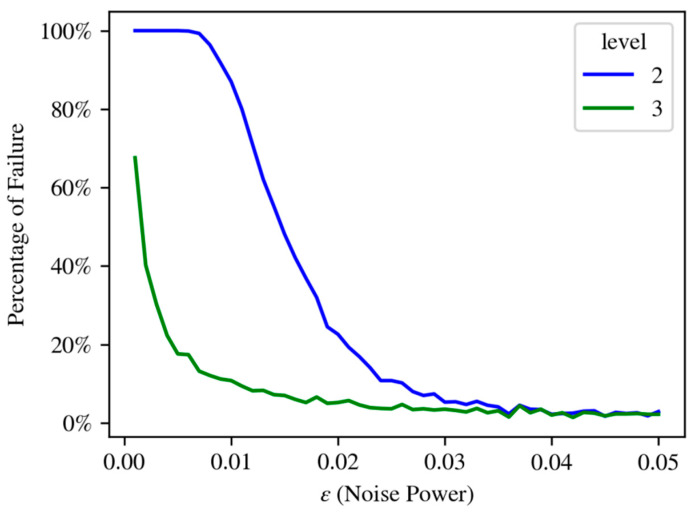
Results of running the configuration of Figure 2 in the GloptiPoly [22] package for PNP. The original configuration failed the second and third levels of the Lasserre hierarchy. Adding uniform noise in [−ε,ε] decreases the percentage of failures, but only when the noise reaches 0.04 (which is about 4% of the data’s average size) does the commonly used solution (level 2) succeed with high probability. Similar results were obtained when using the software package in [23].

The experiments in this paper consisted of obtaining PNP configurations which fail the second and third Lasserre levels, as explained in Lemma 3; such a configuration is depicted in Figure 2. In this figure, the 14 points which were recovered in the optimization described in Section 3.1 are plotted in 3D space, with the corresponding lines of sight for each of them, in the same color. Thus, Figure 2 depicts a setting of the PNP problem which fails the second stage in the Lasserre hierarchy. In order to verify the result, this configuration was run on two existing PNP packages. In addition, the effect of adding noise to the configuration was examined; see Figure 3. This addition of noise was performed in order to demonstrate that the configurations which fail the second stage are “stable”, i.e., do not lie on a low-dimensional manifold in the input space; intuitively speaking, if a configuration fails the second level, so will configurations which are close to it. The noise, which was uniform in the interval [−ε,ε], was added to each of the coordinates of the points $p_{i}$ in the configuration. As the power of the noise increased, the probability of finding a solution in the second level increased, as well as in the third level; since (by definition) the third level solutions contain all the solutions of the second level, the probability for obtaining a solution in the third level is always higher. A more elaborate analysis of the effect of adding noise to the solution is provided in Appendix B.

## 4. Conclusions

We proved, both theoretically and experimentally, that there exist non-degenerate configurations which fail a commonly used convex relaxation to the PNP problem. The probability of failure decreases with the increase of the Lasserre Hierarchy level. We hope that this observation will contribute to further research in solving PNP, as well as other computer vision problems involving convex relaxations. Thus the theoretical implication of the work presented here is that the underlying optimization problem in PNP (and generally in rotation recovery) is difficult in some cases, and cannot always be solved by the first stages of the convex relaxations in the Lasserre hierarchy; however, if the solution in the second level fails, then a practical solution is to continue increasing the level, until a pre-defined threshold is reached. If no solution is obtained, one should resort to his/hers method of choice for constrained polynomial optimization—which are, indeed, more time-consuming than the convex relaxation method.

## Data Availability

The dataset which is relevant (points/lines configuration) can be obtained by mailing the corresponding author.

## Appendix A. The Complexity of Solving the Optimization Problem with Lagrange Multipliers

An alternative approach to minimizing a quartic on *S*^3^ is via the well-known method of Lagrange multipliers, which in this case leads to a system of five equations in five variables ($q_{1},q_{2},q_{3},q_{4}$ and the Lagrange multipliers required for the condition that $\left(q_{1},q_{2},q_{3},q_{4}\right)\in S^{3}$). The degrees of these equations are 3,3,3,3,2. Known bounds for an exact solution are cubic in D, which is defined as the product of the degrees of the equations, multiplied by a factor which is exponential in the number of variables. Another recent bound is $O\left(n{\left(3d\right)}^{3n}\right)$, where n is the number of variables (5 in our case), and *d* the maximal degree (3 in our case). Hence, the complexity of the solution is very high. For a thorough summary of results on the complexity of solving polynomial systems, see [24].

## Appendix B. The Effect of the Noise Power on the Lasserre Levels

As the noise power increases, so does the probability of achieving a solution in a lower Lasserre level. This is due both to the fact that the “pathological” PNP configurations occupy a small volume in the configuration space [6], and to the fact that the solutions obtained in any Lasserre level include those in the lower levels.

A measure to the power of the noise required to obtain an SOS polynomial (which can be solved in any level) is provided by the following lemma:

**Lemma** **A1.**
*Let* $p\left(x_{1}\ldots x_{n}\right)$ *be a form of degree 2d. Then the polynomial* $p\left(x_{1}\ldots x_{n}\right)+r\left(x_{1}^{2d}+\ldots +x_{n}^{2d}\right)$ *, where r is the sum of the absolute values of p’s coefficients, is an SOS.*

**Proof.** A 1891 theorem of Hurwitz states that for any monomial of even degree:

$$x^{\alpha }=x_{1}^{\alpha_{1}}\cdots x_{n}^{\alpha_{n}},\;0\leq \alpha_{k}\in \mathbb{Z},\;\underset{k}{\sum }\alpha_{k}=2d$$

the form:

$$H_{\alpha }\left(x\right)∶=\overset{n}{\underset{k=1}{\sum }}\frac{\alpha_{k}}{2d}x_{k}^{2d}-x_{1}^{\alpha_{1}}\cdots x_{n}^{\alpha_{n}}$$

is SOS. Usually, this is given multiplied by 2d as

$$\overset{n}{\underset{k=1}{\sum }}\alpha_{k}x_{k}^{2d}-2dx_{1}^{\alpha_{1}}\cdots x_{n}^{\alpha_{n}}\;.$$

If any of the $\alpha_{k}$’s is odd, then $x_{k}\mapsto -x_{k}$ shows that

$$H_{\alpha }^{’}\left(x\right)=\overset{n}{\underset{k=1}{\sum }}\frac{\alpha_{k}}{2d}x_{k}^{2d}+x_{1}^{\alpha_{1}}\cdots x_{n}^{\alpha_{n}}$$

is SOS; if all $\alpha_{k}$’s are even, then this expression is a sum of monomial squares. So we can write

$$-x^{\alpha }=H_{\alpha }\left(x\right)-\overset{n}{\underset{k=1}{\sum }}\frac{\alpha_{k}}{2d}x_{k}^{2d},\;x^{\alpha }=H_{\alpha }^{’}\left(x\right)-\overset{n}{\underset{k=1}{\sum }}\frac{\alpha_{k}}{2d}x_{k}^{2d}\;.$$

Suppose now that

$$p\left(x\right)=\underset{\alpha }{\sum }c_{\alpha }x^{\alpha }$$

is any form of degree 2d. Then we may write p as a disjoint sum, depending on whether $c_{\alpha }$ is positive or negative

$$p\left(x\right)=\underset{\alpha }{\sum }\left|c\left(\alpha \right)\right|H_{\alpha }\left(x\right)+\underset{\beta }{\sum }\left|c\left(\beta \right)\right|H{’}_{\beta }\left(x\right)-\overset{n}{\underset{k=1}{\sum }}\left(\underset{\alpha }{\sum }\left|c\left(\alpha \right)\right|\frac{\alpha_{k}}{2d}\right)x^{k}.$$

If we choose

$$r\geq \underset{\alpha }{\sum }\left|c\left(\alpha \right)\right|\geq \underset{\alpha }{\sum }\left|c\left(\alpha \right)\right|\frac{\alpha_{k}}{2d}\;\mathrm{for}\;\mathrm{all}\;k$$

then:

$$p\left(x\right)+r\left(\overset{n}{\underset{k=1}{\sum }}x^{2d}\right)=\underset{\alpha }{\sum }\left|c\left(\alpha \right)\right|H_{\alpha }\left(x\right)+\underset{\beta }{\sum }\left|c\left(\beta \right)\right|H{’}_{\beta }\left(x\right)+\overset{n}{\underset{k=1}{\sum }}t_{k}x^{k}$$

where:

$$t_{k}=r-\underset{\alpha }{\sum }\left|c\left(\alpha \right)\right|\frac{\alpha_{k}}{2d}\;;$$

and so this expression is always an SOS, which completes the proof. □
